# Fusion to Hydrophobin HFBI Improves the Catalytic Performance of a Cytochrome P450 System

**DOI:** 10.3389/fbioe.2016.00057

**Published:** 2016-07-04

**Authors:** Sebastian Schulz, Dominik Schumacher, Daniel Raszkowski, Marco Girhard, Vlada B. Urlacher

**Affiliations:** ^1^Institute of Biochemistry, Heinrich Heine University Düsseldorf, Düsseldorf, Germany

**Keywords:** cytochrome P450 monooxygenase, P450 BM3, CYP102A1, diflavin reductase, fusion protein, hydrophobin, HFBI

## Abstract

Cytochrome P450 monooxygenases (P450) are heme-containing enzymes that oxidize a broad range of substrates in the presence of molecular oxygen and NAD(P)H. For their activity, most P450s rely on one or two redox proteins responsible for the transfer of electrons from the cofactor NAD(P)H to the heme. One of the challenges when using P450s *in vitro*, especially when non-physiological redox proteins are applied, is the inefficient transfer of electrons between the individual proteins resulting in non-productive consumption of NAD(P)H – referred to as uncoupling. Herein, we describe the improvement of the coupling efficiency between a P450 and its redox partner – diflavin reductase – by fusing both enzymes individually to the hydrophobin HFBI – a small self-assembling protein of the fungus *Trichoderma reesei*. The separated monooxygenase (BMO) and reductase (BMR) domains of P450 BM3 from *Bacillus megaterium* were chosen as a P450-reductase model system and individually fused to HFBI. The fusion proteins could be expressed in soluble form in *Escherichia coli*. When HFBI-fused BMO and BMR were mixed *in vitro*, substantially higher coupling efficiencies were measured as compared with the respective non-fused enzymes. Consequently, myristic acid conversion increased up to 20-fold (after 6 h) and 5-fold (after 24 h). Size exclusion chromatography demonstrated that *in vitro* the hydrophobin-fused enzymes build multimeric protein assemblies. Thus, the higher activity is hypothesized to be due to HFBI-mediated self-assembly arranging BMO and BMR in close spatial proximity in aqueous solution.

## Introduction

Cytochromes P450 are heme-containing monooxygenases that catalyze selective oxidations of a vast variety of organic molecules. Among the substrates of P450s are fatty acids, alkanes, steroids, terpenes, and antibiotics (Bernhardt, 1996; Bernhardt and Urlacher, 2014). The significance of these enzymes in biological, pharmacological, and biotechnological processes has been demonstrated in a large number of publications and emphasized in many reviews (Wong, 1998; Guengerich, 2008; Julsing et al., 2008; Jung et al., 2011; Podust and Sherman, 2012; Urlacher and Girhard, 2012; Bernhardt, 2013; Munro et al., 2013).

For their activity, P450s require electrons ultimately derived from the cofactor NAD(P)H. Most P450s are not able to accept these electrons directly, but rely on redox proteins that transfer electrons from NAD(P)H to the heme. Therefore, P450s can generally be considered as multi-protein systems composed of a heme-containing monooxygenase component and either two redox proteins (a ferredoxin or flavodoxin, and a flavin-dependent reductase) or a diflavin cytochrome P450 reductase (Hannemann et al., 2007; McLean et al., 2015). Due to this multi-component nature of P450 systems, their biotechnological applications are mainly restricted to whole-cell processes.

An exceptional group of P450s is represented by the self-sufficient flavocytochromes, in which the heme-containing monooxygenase is naturally fused to its redox partner in one polypeptide chain. This arrangement enables efficient electron transfer within these enzymes resulting in catalytic efficiencies that cannot be matched with other P450 systems (Girvan et al., 2006; McLean et al., 2007). Self-sufficient P450s, therefore, demonstrate in most cases higher catalytic activities among P450 systems (Munro et al., 2013). For example, the highest ever measured turnover rate of a P450 of 17,100 min^−1^ was reported for conversion of arachidonate by the self-sufficient P450 BM3 (CYP102A1) from *Bacillus megaterium* (Noble et al., 1999). In addition, by expression of P450 BM3 on the surface of *Escherichia coli* cells with the Autodisplay system, which allowed several re-uses of the catalyst, a total turnover number of up to 54,700 could be reached – the highest value reported for a P450 to date (Ströhle et al., 2016).

Consequently, one approach to reduce the complexity of P450 multi-protein systems is the construction of “artificial” self-sufficient fusion proteins. A number of mammalian, plant, and bacterial P450s have been fused either to their cognate redox partners or to “foreign” heterologous redox proteins if cognate ones could not be identified [reviewed in Hlavica (2009) and Sadeghi and Gilardi (2013)]. For example, in a recent study, the “artificial” fusion of *Panax ginseng* cytochrome P450-type protopanaxadiol synthase (PPDS) and *Arabidopsis thaliana* NADPH-cytochrome P450 reductase (ATR1) was described (Zhao et al., 2016). When expressed in *Saccharomyces cerevisiae*, the PPDS–ATR1 fusion enzymes conferred a 4.5-fold increase in catalytic activity and 71.1% increase in protopanaxadiol production compared with PPDS and ATR1 co-expression (Zhao et al., 2016). In another recent study, a plant P450 flavonoid 3′-hydroxylase (F3′H), was functionally expressed as a fusion protein with a P450 reductase (CPR) in *E. coli* and successfully employed to produce eriodictyol – a flavonoid with anti-inflammatory and antioxidant activities – from l-tyrosine (Zhu et al., 2014).

However, the generation of artificial fusion proteins is, on the one hand, restricted to an equimolar ratio of redox partner(s):P450 (1:1) and, on the other hand, can significantly restrict the structural flexibility of the individual proteins. Therefore, such artificial fusions do not necessarily end up showing improved catalytic performance (Sibbesen et al., 1996; Cao et al., 2000).

Especially with regard to biocatalytic applications of P450s, in which high catalytic activities are required, efficient P450-reductase interactions are of tremendous importance (Munro et al., 2007; Bernhardt and Urlacher, 2014). In search for a method that allows for site-specific protein targeting and higher flexibility of the partner proteins, an approach called “PUPPET” has been introduced to improve interaction of P450s with redox proteins (Hirakawa and Nagamune, 2010). PUPPET is based on a fusion approach employing the self-assembling heterotrimeric “proliferating cell nuclear antigen” (PCNA) as a scaffold and has been used for P450cam from *Pseudomona putida* and its cognate redox proteins putidaredoxin reductase (PdR) and putidaredoxin (Pdx) (Hirakawa and Nagamune, 2010). Compared with an equimolar mixture of non-fused P450cam:PdR:Pdx *in vitro*, PUPPET displayed a substantially higher catalytic activity, which was explained by improved electron transfer due to the extreme proximity of the P450 and redox partner proteins within the formed complex. However, the trimeric complex dissociated at low concentrations, a problem that was solved by selective introduction of covalent disulfide bridges (Hirakawa et al., 2013). PUPPET is also applicable for P450s whose cognate redox partners are not yet identified, as demonstrated for CYP119 from *Sulfolobus solfataricus* (Suzuki et al., 2014). The PCNA-mediated heterotrimerization of CYP119/PdR/Pdx enhanced CYP119 activity, explained by the authors as a result of high local concentrations of the two redox proteins PdR/Pdx toward CYP119. However, a limitation of PUPPET is that due to the trimeric nature of PCNA, this method is restricted to equimolar concentrations of redox proteins and P450.

It has been demonstrated in many studies that substrate turnovers by P450s were greatly improved by using the electron transfer proteins in molar excess (Girhard et al., 2010; Bell et al., 2012; Cheng et al., 2012; Brill et al., 2014), which is likely due to the higher probability of a P450 to encounter the redox partners in solution. At the same time, the requirement of a high molar excess of redox proteins (e.g., more than 10 times excess of iron–sulfur proteins for class I P450s) is a critical bottleneck in practical P450 applications (Hirakawa et al., 2007).

**Figure 1:**
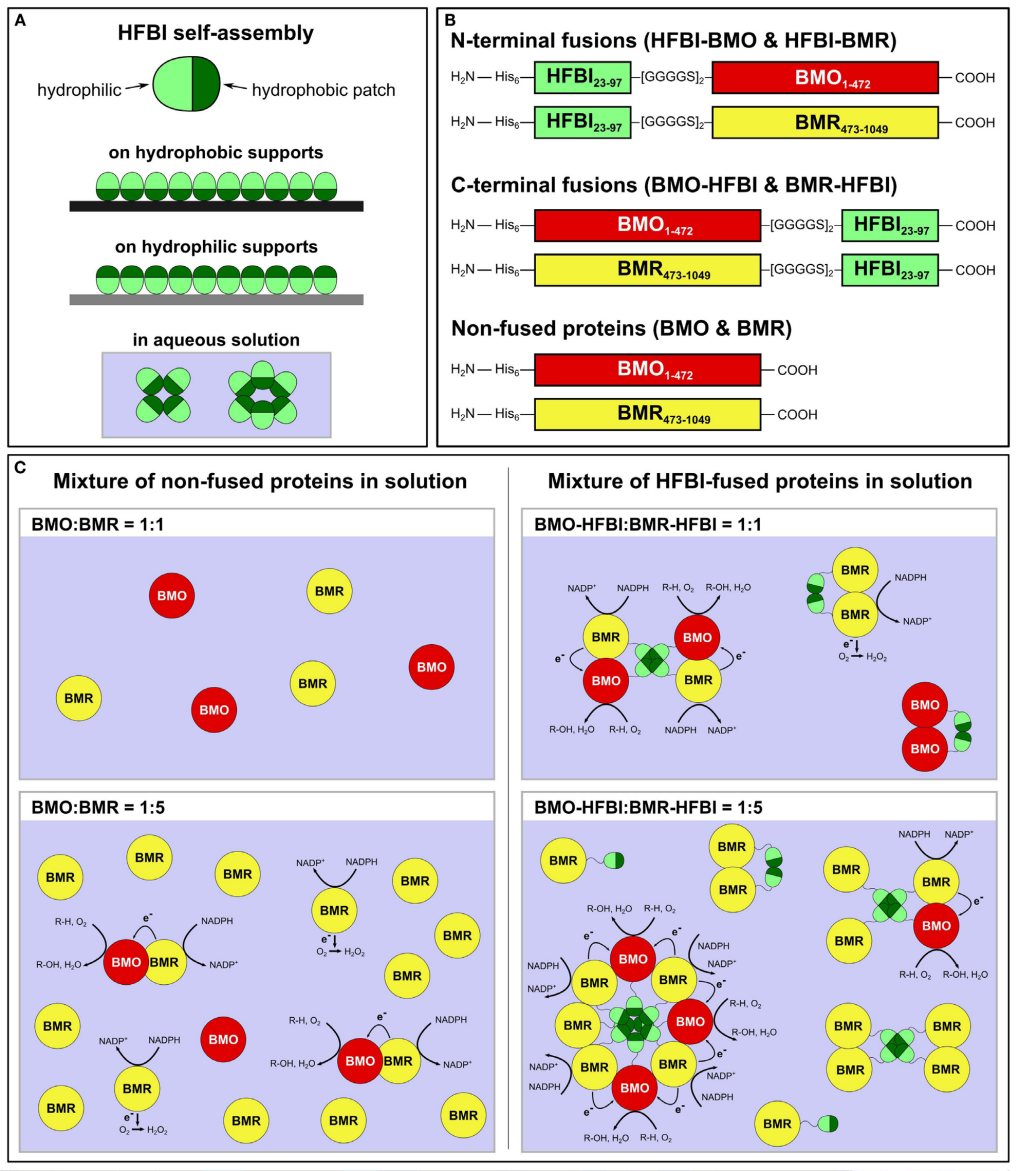
**(A)** Self-assembly of HFBI at hydrophobic or hydrophilic supports reverses the supports’ wettability; self-assembly in aqueous solution is based on hydrophobic–hydrophobic interactions between the hydrophobic patches of HFBI monomers. **(B)** Schematic drawing of HFBI-fused and non-fused proteins. Subscript numerals in ‘BMO’ and ‘BMR’ indicate amino acid positions based on the respective holoenzyme P450 BM3. His_6_: N-terminal hexa-histidine tag. **(C)** Schematic drawings of the BMO/BMR system (the dimensions of the individual proteins are not scaled). Since non-fused BMO and BMR diffuse freely in solution and encounter each other by coincidence (left side), uncoupling reactions result in electrons transferred to O_2_ and lead to formation of reactive oxygen-species (e.g., H_2_O_2_). In the hypothesized HFBI-fusion model (right side), BMO–HFBI and BMR–HFBI are arranged in close spatial proximity by self-assembly of HFBI resulting in reduced uncoupling and improved catalytic performance. The illustrations show octa, tetra, and dimer formation as examples, but other multimeric assemblies are also conceivable. R-H, substrate; R-OH, hydroxylated product.

In view of this requirement, we aimed to establish a novel system that does not underlie the restrictions of equimolar redox protein:P450 concentrations. To achieve this goal, a self-assembling protein is required capable of forming multimers larger than dimers or trimers in solution. We chose hydrophobins, small fungal proteins of about 100 amino acids with a ubiquitous presence of eight cysteine residues forming four disulfide bonds (Wösten and de Vocht, 2000; Bayry et al., 2012). Hydrophobins are amphiphilic molecules comprising a hydrophilic protein part as well as a hydrophobic patch region, which enables these proteins to self-assemble in aqueous solutions as multimers, e.g., tetramers (Szilvay et al., 2006), as well as at hydrophilic–hydrophobic interfaces into organized monolayer membranes (Linder, 2009; Ley et al., 2015) (Figure 1A). The self-assembly in aqueous solution is likely based on hydrophobic–hydrophobic interactions between the hydrophobic patches of hydrophobin monomers (Hakanpää et al., 2006; Szilvay et al., 2006).

The intensively studied hydrophobin HFBI from *Trichoderma reesei* (Nakari-Setälä et al., 1996; Hakanpää et al., 2006) was applied as self-assembling component in our study, as formation of multimers of a fusion protein consisting of an endoglucanase and HFBI in solution has been described and putative decamers have been suggested (Linder et al., 2002).

For this proof-of-concept study, we used the individual domains of P450 BM3 from *B. megaterium*. The holoenzyme P450 BM3 is a natural fusion consisting of a heme-containing monooxygenase (BMO) linked to an FAD-FMN-containing diflavin reductase (BMR) (Narhi and Fulco, 1987). Earlier studies demonstrated that the individual domains can be separated and when reconstituted *in vitro*, activity was observed (Boddupalli et al., 1992a), but at drastically reduced levels as compared with the holoenzyme (4,000 versus 0.5 mol min^−1^ mol_P450_^−1^ measured with palmitic acid for the holoenzyme P450 BM3 versus BMO:BMR reconstituted at a 1:10 ratio, respectively) (Sevrioukova et al., 1997).

Herein, the separated BMO- and BMR domains were individually fused to the hydrophobin HFBI (Figure 1B). The artificial fusion proteins were expressed in *E. coli* and purified. Their combination in different molar ratios *in vitro* resulted in increased coupling efficiencies and substrate conversions. These improvements are hypothesized to originate from hydrophobin-mediated self-assembly between BMO and BMR (Figure 1C).

## Materials and Methods

### Generation of HFBI-Fused Proteins

Plasmids containing the genes coding for BMO_1–472_ and BMR_473–1049_ inserted into pET-28a(+) were generated previously (Girhard et al., 2007; Weber et al., 2010). The subscript numbers refer to the amino acid positions in the holoenzyme P450 BM3 encoded by the *cyp102a1-*gene (GenBank J04832), and are counted including the first methionine.

The open reading frame of the *hfb1*-gene encoding full-length HFBI_1–97_ from *T. reesei* (GenBank Z68124) was codon-optimized for expression in *E. coli* and obtained as synthetic gene from GeneArt (Germany).

Individual genes encoding BMO_1–472_, BMR_473–1049_ and mature HFBI_23–97_ (lacking the N-terminal secretion signal) were amplified by PCR and applied for the generation of HFBI-fused proteins. Primers introduced a sequence encoding the flexible glycine-rich linker [GGGGS]_2_. Details of primer sequences and PCR are given in the Tables S1–S4 in Supplementary Material. Fusion constructs were generated by overlap-extension PCR (oePCR). C-terminal fusions (BMO–HFBI and BMR–HFBI) were generated by a one-step oePCR in which all reaction ingredients were present in the initial reaction mixture. For generation of N-terminal fusion constructs (HFBI–BMO and HFBI–BMR), a two-step oePCR was applied in which the terminal primers were added after the first seven PCR cycles. Forward primers introduced an *Eco*RI and reverse primers a *Xho*I restriction site enabling ligation into the expression vector pET-28a(+). All genes were cloned in pET-28a(+) and carried an N-terminal His_6_-tag. The resulting plasmids were propagated in *E. coli* DH5α and gene sequences were confirmed by Sanger sequencing (GATC-Biotech, Germany).

### Protein Expression

The *E. coli* strain Shuffle T7 Express was used for expression of all enzymes, except for the holoenzyme P450 BM3 that was expressed using strain BL21(DE3) (both strains from New England Biolabs, Germany). Four hundred milliliters of cultures (Lysogeny broth containing 30 μg ml^−1^ kanamycin) were grown at 37°C, 180 rpm on an orbital shaker until the optical density at 600 nm (OD_600_) reached ~0.8. At this point, expression was induced with 100 μM isopropyl β-d-1-thiogalactopyranoside (IPTG). In case of P450 expressions (holoenzyme P450 BM3, BMO–HFBI, HFBI-BMO and BMO), the cultures were supplemented with 80 μg ml^−1^ 5′-aminolevulinic acid and 100 μM FeSO_4_. After induction, protein expression was done at 30°C, 140 rpm. Cells were harvested by centrifugation (18,000× *g*, 30 min, 4°C). The cell pellets were resuspended in 50 mM potassium phosphate buffer (KP_i_), pH 7.5 containing 5% (v/v) glycerol, and 100 μM phenylmethylsulfonyl fluoride (PMSF), disrupted by sonication on ice (3 × 1.5 min, 1 min intermission), and cell debris was removed by centrifugation (49,000× *g*, 30 min, 4°C). The supernatants (cleared cell lysates) were recovered and stored at −20°C until purification.

### Protein Purification, SDS-PAGE, and Immunodetection

Protein purification was done by immobilized metal ion affinity chromatography (IMAC) using a HisTrap FF crude column (2 × 5 ml) on an ÄKTApurifier system (GE Healthcare, Germany). The column was equilibrated with 50 mM KP_i_, pH 7.5, 500 mM NaCl prior to loading of the protein sample. Non-specifically bound proteins were washed off the column with three column volumes (CV) of the same buffer and five CV of the buffer containing 7.5–25 mM imidazole. The protein of interest was eluted with buffer containing 200 mM imidazole.

Purified proteins were desalted with a HiPrep 26/10 desalting column (GE Healthcare) and eluted in 50 mM KP_i_, pH 7.5, 25 mM NaCl, 5% (v/v) glycerol. Protein samples were concentrated by ultrafiltration (30 kDa molecular weight cutoff) and stored at −20°C.

Proteins were visualized by SDS-PAGE (Laemmli, 1970) as well as Western blotting in combination with immunodetection using an antibody recognizing the His_6_-tag epitope. Semi-dry transfer of proteins onto nitrocellulose membranes was conducted with 2 mA/cm^2^ for 30 min. Membranes were blocked with TBST [50 mM TrisHCl, pH 7.5, 150 mM NaCl, 0.1% (v/v) Tween 20] containing 1% (w/v) skimmed milk powder for 1 h and incubated for another 1.5 h with an Anti-His_6_-peroxidase conjugate (Anti-His_6_-peroxidase mouse IgG, Roche Diagnostics, Germany) according to the manufacturer’s instructions. After three washing steps with TBST, the precipitating BM Blue POD substrate (Roche Diagnostics) was applied for immunodetection following the supplier’s instructions.

### Size Exclusion Chromatography

Assembly of BMO–HFBI/BMR–HFBI was analyzed by size exclusion chromatography (SEC) on a 16/60 Superdex 200 prep grade column (GE Healthcare) with a bed volume of 120 ml and a separation range of 10–600 kDa. The column was equilibrated with 3 CV of 50 mM KP_i_, pH 7.5, 5% (v/v) glycerol prior to loading 1 ml of protein samples. Samples containing an equimolar mixture of BMR-HFBI and BMO-HFBI (53 μM each) were incubated for 30 min at 30°C prior to loading. Proteins were eluted at a flow rate of 0.5 ml min^−1^ in 1.5 CV buffer and detected by measuring the absorbance at 280 nm. To estimate molecular weights, the standard proteins apoferritin (equine spleen; 443 kDa), β-amylase (potato; 200 kDa), alcohol dehydrogenase (yeast; 150 kDa), albumin (bovine serum; 66 kDa), and carbonic anhydrase (bovine erythrocytes; 29 kDa) (all from Sigma-Aldrich, Germany) were used, whereas the exclusion limit of the column (≥ 600 kDa) was determined by using blue dextran (elution volume 40.8 ml).

### Determination of Protein Concentrations

The concentrations of diflavin reductase containing enzymes were calculated from the absorbance at 452 and 466 nm with ε_452_ = 10 mM^−1^ cm^−1^ and ε_466_ = 10 mM^−1^ cm^−1^ (Sevrioukova et al., 1996). Concentrations of P450 containing enzymes were calculated from CO-difference spectra with ε_450-490_ = 91 mM^−1^ cm^−1^ as described elsewhere (Omura and Sato, 1964b).

Total protein concentrations were determined using the method described by Bradford (1976) and bovine serum albumin as standard.

### Cytochrome *c* Assay

Cytochrome *c* reducing activities of reductases were determined in 96-microwell plates in total volumes of 200 μl containing 50 mM KP_i_, pH 7.5, and 100 μM cytochrome *c* from equine heart (Sigma-Aldrich). Reactions were started by addition of 100 μM NADPH. The increase of absorbance at 550 nm was followed and cytochrome *c* reducing activities were calculated using ε_550_ = 21 mM^−1^ cm^−1^. Specific activities are given in U mg^−1^ of total protein, where 1 U is defined as the amount of enzyme catalyzing the reduction of 1 μmol cytochrome *c* per minute. Cytochrome *c* reducing activities of soluble protein fractions obtained from *E. coli* cells lacking expressed heterologous reductase were subtracted from the results.

### Bioconversion of Myristic Acid and Product Analysis

Conversions of myristic acid were performed at 30°C in a total reaction volume of 300 μl. Samples contained 50 mM KP_i_, pH 7.5, 1 mM MgCl_2_, 2% (v/v) DMSO, 200 μM myristic acid, 4 mM d-glucose 6-phosphate, and 3 U glucose 6-phosphate dehydrogenase from *Leuconostoc mesenteroides* (Sigma-Aldrich) for cofactor regeneration, 600 U catalase from bovine liver (Sigma-Aldrich) to decompose hydrogen peroxide that can be produced within the P450 catalytic cycle, 200 μM NADPH, 1 μM P450 (BMO–HFBI or BMO), and 1 or 5 μM reductase (BMR–HFBI or BMR). Additionally, a reaction was set up that contained 1 μM holoenzyme P450 BM3 instead of BMO and BMR.

Analysis of reaction products was done by gas chromatography coupled with mass spectrometry (GC/MS). Samples were acidified with HCl, extracted with diethyl ether, derivatized with *N*,*O*-bis(trimethylsilyl)trifluoroacetamide containing 1% trimethylchlorosilane, and analyzed, as described previously (Girhard et al., 2013).

### Measurement of NADPH Oxidation Rates and Coupling Efficiencies

NADPH oxidation rates as well as coupling efficiencies were determined in a total reaction volume of 1 ml. Samples contained 50 mM KP_i_, pH 7.5, 2% (v/v) DMSO, 200 μM myristic acid, 1 μM P450 (BMO–HFBI or BMO), and 1 or 5 μM reductase (BMR–HFBI or BMR). Additionally, a reaction was set up that contained 1 μM holoenzyme P450 BM3 instead of BMO and BMR.

Reactions were started by addition of 200 μM NADPH and the absorbance decrease at 340 nm was followed. NADPH oxidation rates were calculated using ε_340_ = 6.22 mM^−1^ cm^−1^ and related to the applied amount of P450 (nmol min^−1^ nmol_P450_^−1^).

After NADPH was completely consumed, the samples were prepared for GC/MS analysis as described above. The coupling efficiencies were calculated as the ratio between the concentrations of product formed and NADPH consumed.

## Results

### Expression and Characterization of HFBI-Fused Proteins

The two domains of P450 BM3, the monooxygenase domain BMO and the diflavin reductase domain BMR were separated and fused individually to hydrophobin HFBI. Fusion proteins were constructed using the mature form of hydrophobin HFBI lacking its N-terminal 22 amino acids-long secretion signal sequence. HFBI was fused to BMO and BMR in two orientations: either at their N-termini (designated as HFBI–BMO and HFBI–BMR) or at their C-termini (designated as BMO–HFBI and BMR–HFBI) (Figure 1B). We inserted a flexible [GGGGS]_2_ linker, because this linker type is frequently used for the construction of fusion proteins allowing for motility of separate domains (Lu and Feng, 2008; Gruner et al., 2012; Chen et al., 2013; Bakkes et al., 2015). Due to the large differences in molecular weights of HFBI (7.5 kDa) and BMO or BMR (54 and 64 kDa, respectively), a flexible but not too long linker was considered suitable to retain the functional integrity of both domains; especially the self-assembling capability of the rather small HFBI domain should not be superposed by the larger BMO or BMR domains. The separated non-fused BMO and BMR domains served as reference proteins for comparison of biochemical and biocatalytic properties.

The four disulfide bridges present in hydrophobins stabilize their structure as described for HFBII (Hakanpää et al., 2004). As HFBI possesses a very similar overall structure (Hakanpää et al., 2006), we used the *E. coli* Shuffle T7 Express strain optimized for functional expression of proteins bearing disulfide bridges to express the HFBI fusion proteins. The same strain has also been applied for the expression of other HFBI fusion proteins earlier (Gruner et al., 2012).

**Figure 2:**
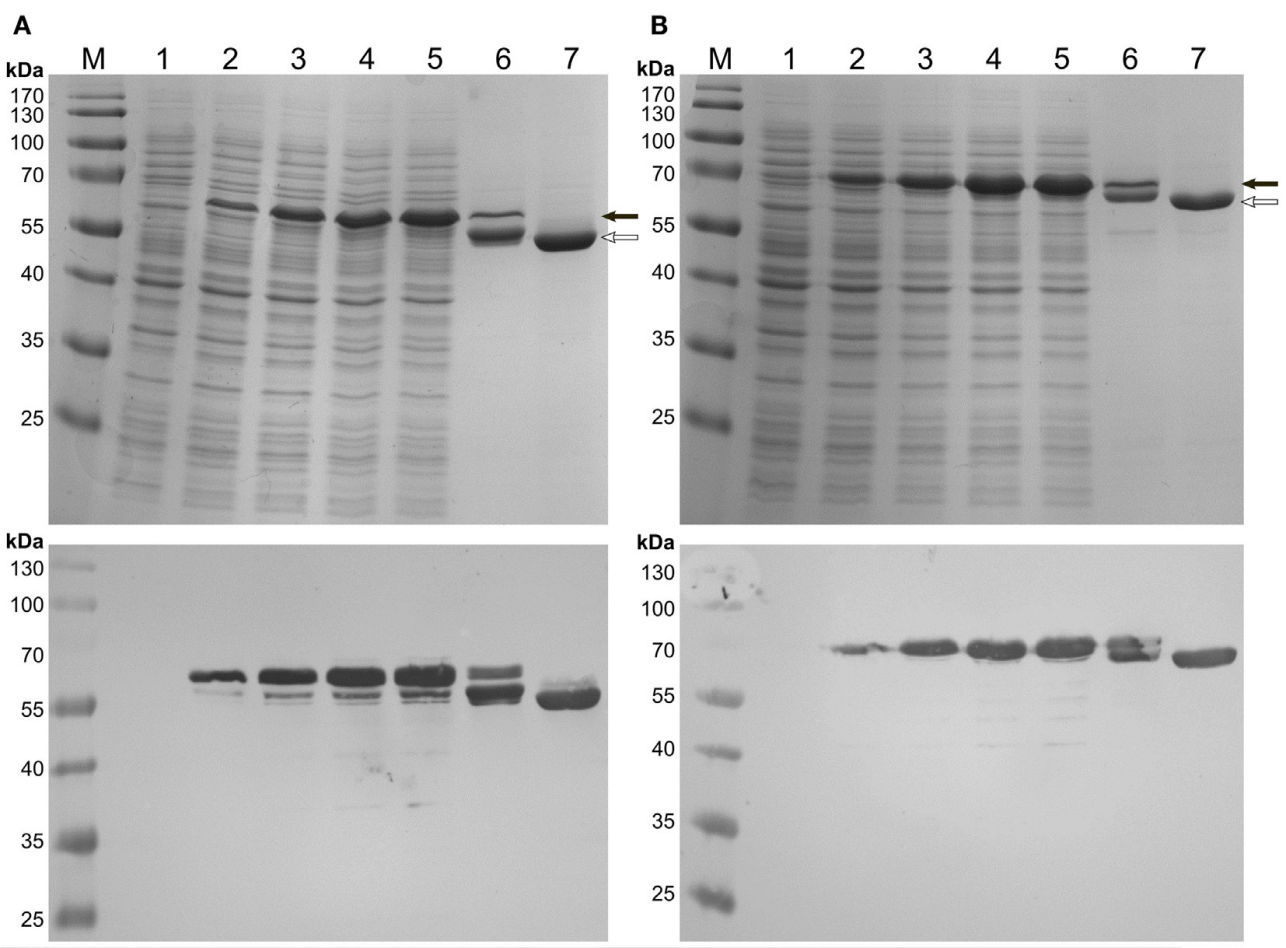
**SDS-PAGE (top pictures) and immunodetection (bottom pictures) of C-terminal HFBI-fusion proteins expressed in *E. coli* Shuffle T7 Express**. **(A)** BMO–HFBI and **(B)** BMR–HFBI. (1) Whole-cells before induction; (2) cells 1 h post-induction; (3) cells 2 h post-induction; (4) cells 3 h post-induction; (5) cells 4 h post-induction; (6) purified BMO–HFBI or BMR–HFBI of cells harvested 18 h post-induction; (7) purified non-fused BMR or BMO of cells harvested 18 h post-induction. BMO–HFBI (66 kDa) and BMR–HFBI (76 kDa) are marked with a full arrow. Non-fused BMO (57 kDa) and BMR (68 kDa) are marked with an open arrow. Approximately 10 μg total protein per lane. M, molecular weight marker.

Expression of the C-terminal HFBI fusions, BMO–HFBI (Figure 2A) and BMR–HFBI (Figure 2B) resulted in adequate amounts of soluble protein. The N-terminal fusions HFBI–BMO and HFBI–BMR were expressed at lower levels compared with the C-terminal fusions. Western blot analysis indicated only a slight soluble expression of HFBI–BMO (~30%) which was, however, entirely absent in case of HFBI–BMR with all protein detected as insoluble (Figures S1 and S2 in Supplementary Material).

Non-fused BMO was almost exclusively found soluble, whereas ~50% of non-fused BMR built insoluble aggregates during expression (Figure S3 in Supplementary Material).

Soluble protein fractions bearing the heme-containing BMO domain were analyzed by recording CO-difference spectra that provides a measure of functional P450 based on correctly coordinated heme (Omura and Sato, 1964a,b). Generally, the observations from SDS-PAGE and immunodetection were in good agreement with the measurement of functional P450 in the soluble protein fractions. The C-terminal BMO–HFBI fusion was expressed at 650 nmol l^−1^, whereas no correctly folded P450 was detectable in the samples with the N-terminal HFBI–BMO fusion. For comparison, the non-fused BMO was expressed at a concentration of 1,100 nmol l^−1^ (Table 1).

**Table 1:** **Expression of BMO-enzymes and cytochrome *c* reducing activities of BMR-enzymes (soluble protein fractions after cell lysis)**.

Enzyme	Expression (nmol l^−1^)	Cytochrome *c* activity (U mg^−1^)
BMO–HFBI	650	–
HFBI–BMO	n.d.	–
BMO	1,100	–
BMR–HFBI	–	0.88
HFBI–BMR	–	0.03
BMR	–	1.04

*n.d., not detectable; –, not measured*.

Soluble protein fractions containing the BMR domain were assessed for their ability to reduce cytochrome *c*. Samples containing the C-terminal BMR–HFBI fusion displayed a 29-fold higher cytochrome *c* reducing activity compared with that of the N-terminal HFBI–BMR fusion, which showed a very low activity of 0.03 U mg^−1^. Non-fused BMR displayed a slightly higher activity compared with that of BMR–HFBI (Table 1).

Since adequate expression and activity were obtained for the C-terminal fusion proteins only, BMO–HFBI and BMR–HFBI were selected for all further experiments.

After IMAC-purification, SDS-PAGE of BMO–HFBI and BMR–HFBI revealed protein bands of the expected molecular masses. However, an additional band migrating slightly below the expected one was visible for both fusions. Both bands remained visible on a Western blot (Figure 2). As immunodetection was done using an antibody directed against the His_6_-tag epitope located on the N-terminus, we suggest that the degradation of fusion proteins occurred from their C-termini. If expressions were conducted for longer time periods of 18 h, the degradation was even more pronounced yielding higher ratios of truncated than intact fusion proteins. Therefore, the expression times were shortened to 2–6 h to ensure high yields of functional full-length fusion proteins.

**Figure 3:**
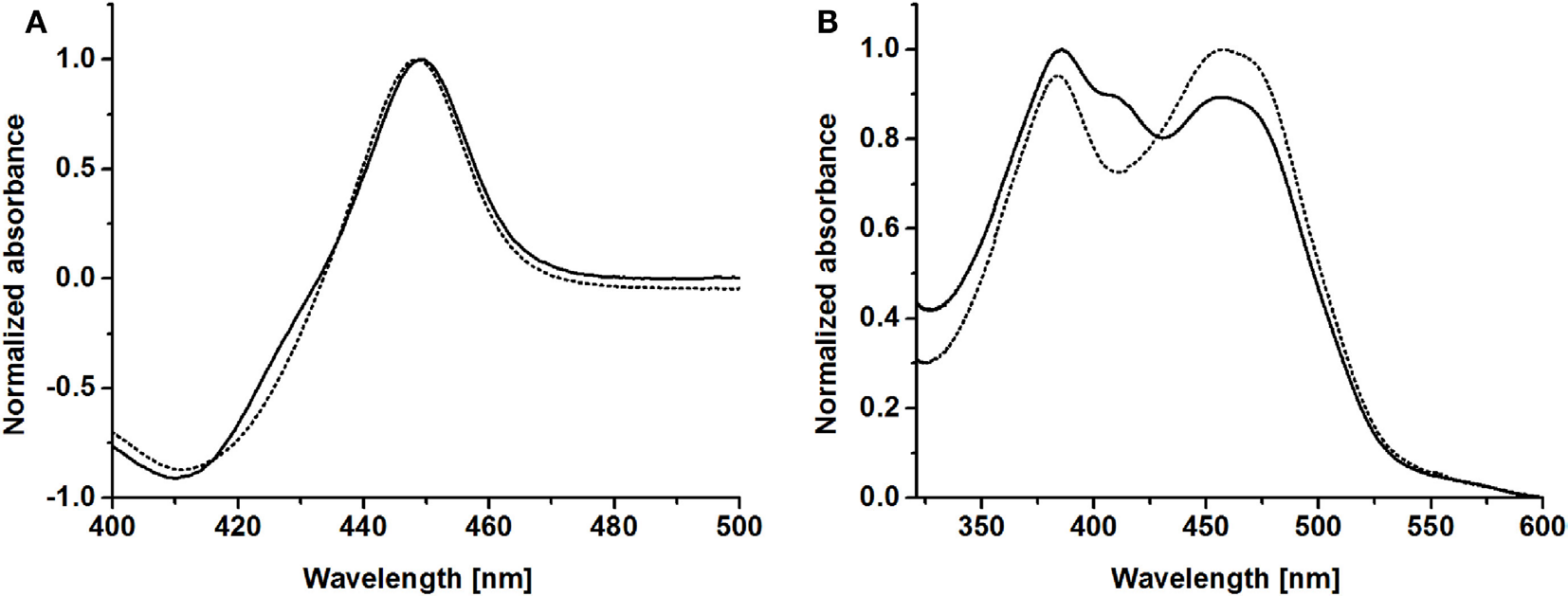
**Spectral properties of HFBI-fused and non-fused BMO and BMR**. **(A)** CO-difference spectra of purified BMO–HFBI (solid line) and BMO (dotted line). **(B)** Absorbance spectra of purified BMR–HFBI (solid line) and BMR (dotted line).

CO-difference spectra of purified BMO–HFBI and BMO showed the characteristic P450 absorbance maximum around 450 nm, indicating correctly coordinated heme; specifically, the absorbance maximum was observed at 449 nm for both proteins (Figure 3A). Absorbance spectra of purified BMR showed maxima at 383 and 457 nm, which is in good agreement with literature data reporting maxima at 385 and 456 nm (Neeli et al., 2005). Values for purified BMR–HFBI were very similar with maxima at 385 and 457 nm (Figure 3B). Both spectra indicate the presence of two prosthetic groups, namely FAD and FMN. In contrast to BMR, however, in the BMR–HFBI spectrum the 457 nm maximum was less pronounced compared with the 385 nm maximum and had an additional plateau around 403–411 nm.

### Myristic Acid Conversion by HFBI-Fused Proteins

To assess the activity of HFBI-fused enzymes, myristic acid – a well-studied substrate of the holoenzyme P450 BM3 (Boddupalli et al., 1992b; Whitehouse et al., 2012) – was chosen as model substrate. The holoenzyme P450 BM3 catalyzes the oxidation of myristic acid at subterminal positions to give predominantly the ω-1, ω-2, and ω-3 hydroxylated products (Figure S4 in Supplementary Material).

**Figure 4:**
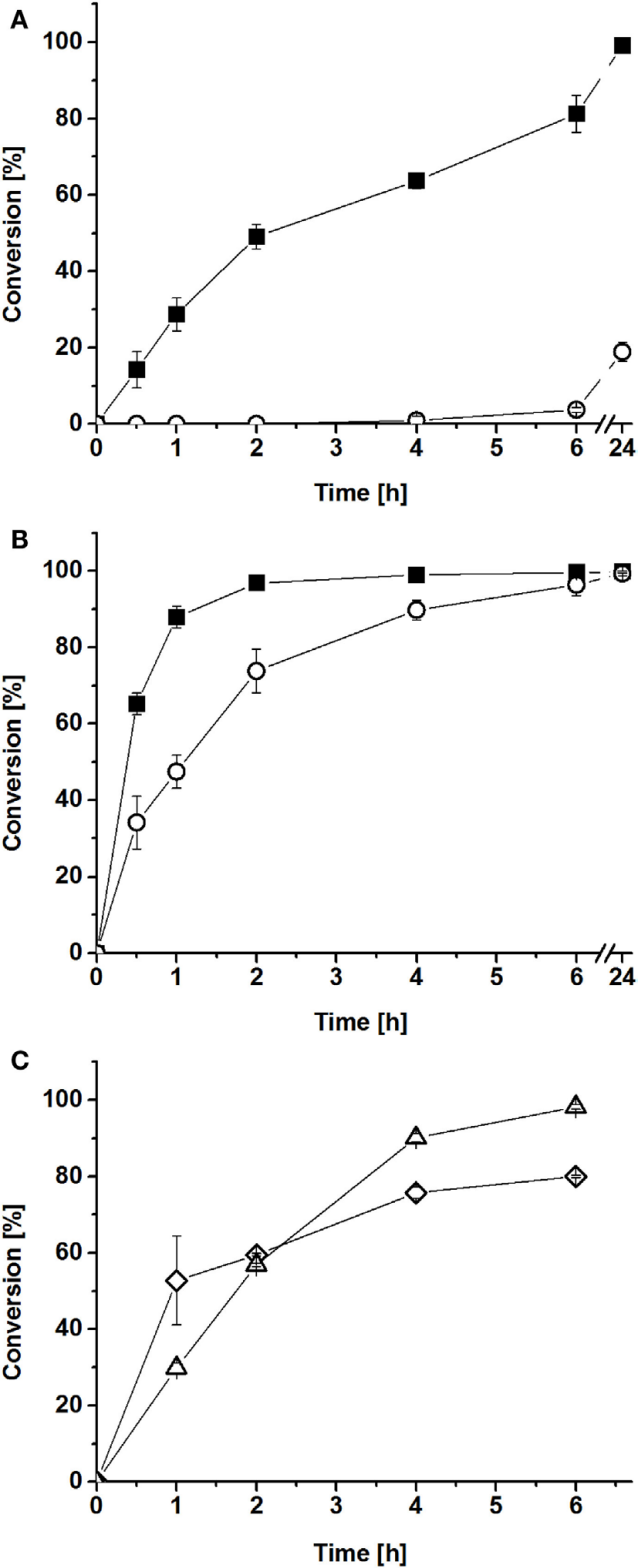
**Conversions of myristic acid by reconstituted P450/reductase systems *in vitro***. BMO–HFBI/BMR–HFBI (filled squares) in comparison to BMO/BMR (open circles) **(A)** equimolar ratios (1:1 μM). **(B)** Fivefold molar excess of reductase (1:5 μM). Mean ± SD (*n* = 3). **(C)** “Cross-conversion” experiment with a HFBI-fused and a corresponding non-fused protein. BMO/BMR–HFBI (open diamonds) or BMO–HFBI/BMR (open triangles), both with fivefold molar excess of the reductase (1:5 μM). Mean ± SD (*n* = 2).

In the first experiment, hydrophobin-fused BMO–HFBI and BMR–HFBI were mixed *in vitro* in equimolar ratio and conversion of myristic acid was compared with those of non-fused BMO and BMR. Conversion with BMO–HFBI/BMR–HFBI achieved 78 and 99% after 6 and 24 h, whereas for BMO/BMR only 3.9 and 19% conversion was observed within these periods of time, respectively (Figure 4A).

As described in the Section “Introduction,” P450 systems can generally be improved by using the electron transfer proteins in a molar excess (Girhard et al., 2010; Bell et al., 2012; Cheng et al., 2012; Brill et al., 2014). Thus, in a second experiment, myristic acid conversion was assayed with fivefold molar excess of BMR–HFBI over BMO–HFBI. In this case, conversion with BMO–HFBI/BMR–HFBI was almost complete after 2 h (97%), whereas reactions with BMO/BMR required 6 h to convert the same amount of myristic acid (Figure 4B). When comparing the reaction system with equimolar P450:reductase ratios to those employing fivefold excess of reductase, it can be stated that the improvement of the system by fusion to HFBI was more pronounced if the proteins were applied in equimolar ratio.

Next, we aimed to investigate whether the observed improvements in myristic acid conversion could also be achieved if only one of the partners was fused to HFBI. Hence, “cross-conversion” experiments were performed, in which a HFBI-fused protein was combined with the corresponding non-fused partner [(i) BMO/BMR–HFBI and (ii) BMO–HFBI/BMR], and were conducted using a fivefold molar excess of the reductase. In these cases, the conversions of myristic acid reached 57–60% after 2 h (Figure 4C), which is lower compared with the 97% reached with both partners fused to HFBI (BMO–HFBI/BMR–HFBI), and interestingly also slightly lower compared with the reaction with non-fused BMO/BMR (74%).

For all reconstituted systems predominantly ω-1 hydroxylated myristic acid and similar ratios of ω-2 and ω-3 hydroxylated products were observed, which is in agreement with the literature data for the holoenzyme P450 BM3 (Boddupalli et al., 1992b; Whitehouse et al., 2012). The product distributions of both fused and non-fused systems were overall similar; thus, the fusion of HFBI did not affect the regioselectivity of BMO (Table S5 in Supplementary Material).

### Catalytic Performance of HFBI-Fused Proteins

The HFBI-fused proteins were analyzed in more detail regarding NADPH oxidation rates and coupling efficiencies during myristic acid hydroxylation (Table 2). When BMO–HFBI/BMR–HFBI were applied in equimolar ratio, an NADPH oxidation rate of 4.2 nmol min^−1^ nmol_P450_^−1^ was measured, and the coupling efficiency between NADPH consumed and product formed reached 57%. A fivefold molar excess of BMR–HFBI resulted in a sixfold increased NADPH oxidation rate (27.0 nmol min^−1^ nmol_P450_^−1^) compared with the equimolar reconstituted HFBI-system. Strikingly, the coupling efficiency was increased to 93%.

**Table 2:** **Catalytic performance of reconstituted P450-reductase systems**.

Enzymes	Ratio P450:Reductase	NADPH oxidation rate^a^	Coupling efficiency (%)
BMO–HFBI/BMR–HFBI	1:1	4.2 ± 0.1	57.4 ± 16.6
BMO/BMR	1:1	n.d.^b^	–^c^
BMO–HFBI/BMR–HFBI	1:5	27.0 ± 7.2	92.7 ± 4.8
BMO/BMR	1:5	26.2 ± 9.8	65.2 ± 16.3
Holoenzyme P450 BM3		368.6 ± 52.3	91 ± 1.1

*^a^Initial NADPH oxidation rate (nmol min^−1^ nmol_P450_^−1^)*.

*^b^Not detectable (NADPH consumption and substrate conversion were below the detection limits)*.

*^c^Due to non-detectable NADPH consumption and substrate conversion, the coupling efficiency could not be determined*.

In comparison, for the equimolar mixture of non-fused BMO/BMR, the NADPH oxidation rate and coupling efficiency were too low to be quantified. For the 1:5 mixture of BMO/BMR, the NADPH oxidation rate reached 26.2 nmol min^−1^ nmol_P450_^−1^, which is comparable to those of the fused enzymes; however, a much lower coupling efficiency was measured in this case (Table 2).

The NADPH consumption rate of the holoenzyme P450 BM3 was still 14-fold higher (368.6 nmol min^−1^ nmol_P450_^−1^) than that of the HFBI-fused system with fivefold excess of reductase and 100% conversion was achieved already within the first 5 min of the reaction (data not shown), but remarkably, the coupling efficiency was about the same (91%).

The myristic acid conversions obtained after 2 h with cofactor regeneration (Figure 4) were in good agreement with the obtained coupling efficiencies of the reconstituted systems. The highest conversion of 97% was achieved with BMO–HFBI/BMR–HFBI with fivefold excess of reductase (Figure 4B). For this system, accordingly the highest coupling efficiency was determined. For equimolar reconstituted systems, conversion after 2 h was only detectable in reactions with BMO–HFBI/BMR–HFBI (49%) which was accompanied with measurable NADPH oxidation rates and coupling efficiency. By contrast, catalytic parameters and conversion were not detectable when non-fused BMO/BMR was reconstituted in equimolar 1:1 ratio.

### Size Exclusion Chromatography Experiments

**Figure 5:**
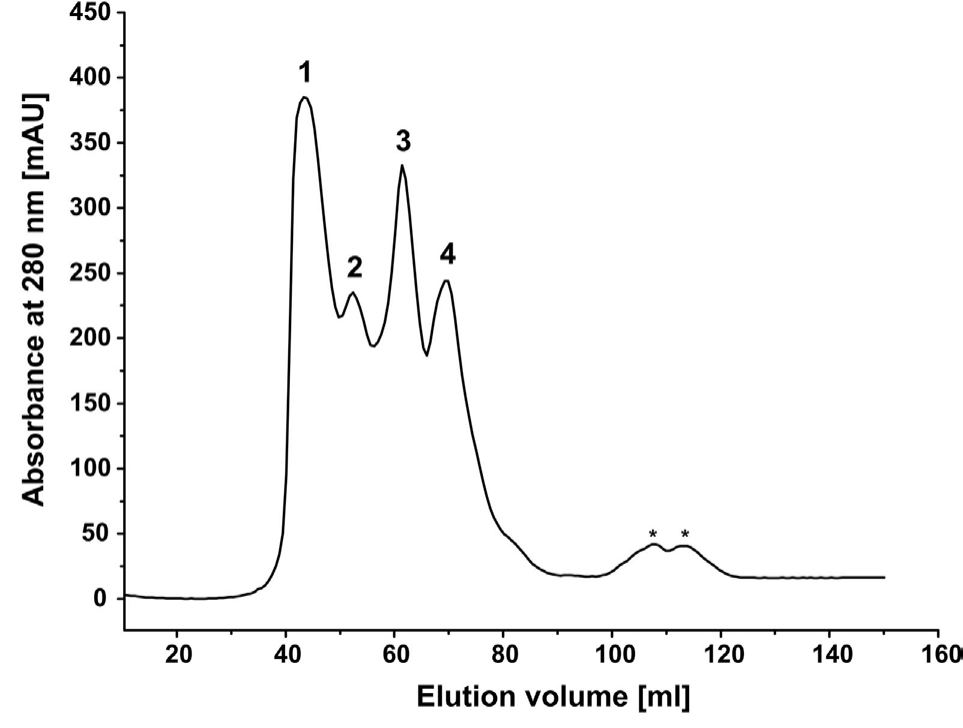
**Size exclusion chromatography of a mixture of BMO-HFBI:BMR–HFBI in equimolar ratio (53 μM each)**. The void volume of the column was 40.8 ml (≥600 kDa). Molecular weights were estimated using a linear calibration curve with standard proteins (for details refer to Section “Materials and Methods”). Peak 1, ca. 530 kDa (43.6 ml); Peak 2, 276 kDa (52.5 ml); Peak 3, 143 kDa (61.4 ml); Peak 4, 78 kDa (69.6 ml). Peaks eluting between 100 and 120 ml (designated with asterisks) denote molecular weights smaller than 29 kDa and likely represent degradation products.

We hypothesized that, in aqueous solutions, the self-assembling capability of hydrophobins arranges the monooxygenase and reductase domains in close spatial proximity, consequently increasing the catalytic performance of the reconstituted system. Indeed, the HFBI-fused enzymes displayed improved catalytic performance compared with the non-fused enzymes independently on the molar ratio employed. To gain a first hint, if this improvement is in fact due to self-assembling of the HFBI-fusion proteins, an equimolar mixture of BMO–HFBI/BMR–HFBI (66 and 76 kDa molecular weight of monomers) was analyzed by SEC (Figure 5). The elution profile of the HFBI-fused enzymes showed multiple peaks, which is indicative of a heterogeneous protein population. The largest peak eluted at 43.6 ml, which corresponds to a molecular weight of ~530 kDa. The high-molecular weight species are multimeric assemblies; here, putative hexa- to octameric complexes are conceivable. Moreover, additional peaks were detected corresponding to species with an apparent molecular weight of 276 kDa (putative tetramer), 143 kDa (putative dimer), and 78 kDa (likely the monomers).

Although the exact compositions of the different multimeric complexes formed by HFBI-fused enzymes – including the stoichiometry in which the individual BMO and BMR components are present – remained unidentified, the presence of octa- and other multimers gives a good hint that the HFBI-fused proteins are able to self-assemble in solution.

## Discussion

The capability of hydrophobin HFBI to self-assemble was analyzed in this study for its potential to improve the catalytic performance of a reconstituted *in vitro* system consisting of a P450 monooxygenase and its corresponding diflavin reductase. Therefore, the HFBI moiety was fused individually to BMO and BMR.

It has been described previously that expression of hydrophobins in *E. coli* often leads to formation of inclusion bodies (Espino-Rammer et al., 2013), which also accounts for derived fusion proteins (Morris et al., 2013). Isolation from insoluble protein fractions is conducted with denaturing agents followed by oxidative refolding (Morris et al., 2013). Within this study, expression of soluble and functional proteins was only possible in case of C-terminal HFBI-fusion (BMO–HFBI and BMR–HFBI). By contrast, expressions of fusion proteins bearing HFBI at their N-termini yielded largely insoluble and non-functional proteins. A similar expression strategy was employed using glutathione *S*-transferase (GST) as N-terminal fusion partner leading to soluble expression of GST-hydrophobin fusion proteins. GST was even necessary to keep the class II hydrophobins HFBIV and HFBVII in solution in *in vitro* experiments (Espino-Rammer et al., 2013). Similarly, Morris et al. (2011) reported soluble expression of the class I hydrophobin DewA from *Aspergillus nidulans*, which was produced as a fusion protein with N-terminally located human ubiquitin. In accordance to the literature, our data indicate that soluble expression of HFBI in *E. coli* is favored by fusion of a highly soluble large protein (like BMO or BMR) to the N-terminus of HFBI.

Spectra of purified BMO–HFBI and BMR–HFBI were very similar to that of their non-fused counterparts BMO and BMR. The CO-difference spectrum of BMO–HFBI indicated a proper incorporation of the heme group during expression of the fusion. The absorbance spectrum of BMR–HFBI showed two specific maxima indicative for FAD–FMN-bound diflavin reductases (Sevrioukova et al., 1996; Neeli et al., 2005). The ratios of maxima between BMR–HFBI and BMR were different, and BMR–HFBI showed an additional shoulder at 403–411 nm. The observed differences are probably due to the fact that HFBI was fused to the C-terminus of BMR near the FAD coordination site. Similar albeit less pronounced observations were made previously in mutagenesis studies focusing on the NADPH-binding FAD-region of BMR. In that case, the ratios between shorter and longer wavelength maxima were also changed which was explained by the exposure of FAD to the solvent (Neeli et al., 2005). The shoulder at 403–411 nm might be explained by the presence of a certain fraction of anionic FAD^−^ radicals (Müller et al., 2014).

We observed that with increasing expression time HFBI-fused proteins were not entirely expressed in intact full-length form. Degradation products derived from C-terminal truncation were frequently observed at longer expression times. On SDS-gels, degradation products of BMO–HFBI and BMR–HFBI migrated approximately at molecular masses comparable to those of non-fused BMO and BMR, respectively. This is likely the result of cleavage of the glycine-serine linker leading to HFBI-deficient protein subspecies. Gycine-rich linkers were previously found to be prone to proteolytic cleavage by *E. coli* peptidases (Kavoosi et al., 2007) which might provide an explanation for our observation. Furthermore, degradation of fusion proteins bearing the hydrophobin DewA were described as well (Wohlleben et al., 2010). A systematic optimization of the linker sequence between both fusion partners, e.g., by the recently described “DuaLinX” method (Bakkes et al., 2015), may lead to enhanced stability of the fusion proteins.

Catalytic activity of the BMO–HFBI/BMR–HFBI system was higher as compared with that of the respective non-fused BMO/BMR system. The catalytic performance was assessed in two ways: (i) time-dependent substrate conversion using cofactor regeneration and (ii) the assessment of catalytic parameters of reconstituted systems by measurements of initial rates under cofactor limiting conditions. Both methods independently confirmed a beneficial effect of the HFBI-fusion on catalytic performance. This effect was more pronounced when reconstituted in equimolar ratio, but also visible at a 1:5 ratio of BMO-HFBI:BMR-HFBI. Furthermore, fusion to HFBI did not affect the regioselectivity of BMO in myristic acid hydroxylation.

Remarkably, the coupling efficiency of the BMO–HFBI/BMR–HFBI reaction reached up to 93%, which is as good as that of the holoenzyme P450 BM3. This improvement is hypothesized to originate from more efficient electron transfer from BMR to BMO as result of HFBI-mediated assembly of the proteins in close spatial arrangement in solution. Our SEC data support this hypothesis, since high-molecular weight complexes were observed for equimolar mixtures of BMO–HFBI/BMR–HFBI. The elucidation of the exact nature of these multimeric complexes, however, requires further studies.

In conclusion, the presented approach employing the hydrophobin HFBI to mediate P450-reductase interaction represents a promising tool for the improvement of the catalytic performance of multi-component P450 systems. Since redox partners’ ratios can be flexibly adjusted, the system can be fine-tuned according to the needs of individual P450s. The hydrophobin fusion approach might also be extended to other multi-enzyme systems where arrangement of the individual enzymes in close spatial proximity is a prerequisite to achieve high catalytic activities.

## Author Contributions

VU, MG, and SS contributed to the conception of the work. DS, DR, and SS produced the data of the work, whereas all authors contributed to data analysis and interpretation. SS drafted the manuscript. All authors revised the manuscript critically and gave final approval of the version to be published.

## Conflict of Interest Statement

The authors declare that the research was conducted in the absence of any commercial or financial relationships that could be construed as a potential conflict of interest.
