# Supplementary material for: Fusion to Hydrophobin HFBI Improves the Catalytic Performance of a Cytochrome P450 System
Source: Front Bioeng Biotechnol. 2016 Jul 4;4:57. doi: 10.3389/fbioe.2016.00057 (PMC4930934; doi:10.3389/fbioe.2016.00057)
Supplement: Supplementary file 1 [file Data_Sheet_1.PDF]

Supplementary Material

Fusion to hydrophobin HFBI improves the catalytic performance of a cytochrome P450 system

Sebastian Schulz, Dominik Schumacher, Daniel Raszkowski, Marco Girhard, Vlada B. Urlacher*

*** Correspondence:** Vlada B. Urlacher: vlada.urlacher@uni-duesseldorf.de

1 Supplementary Figures and Tables

1.1 Supplementary Figures

1.1.1 Expression of HFBI-BMO and HFBI-BMR

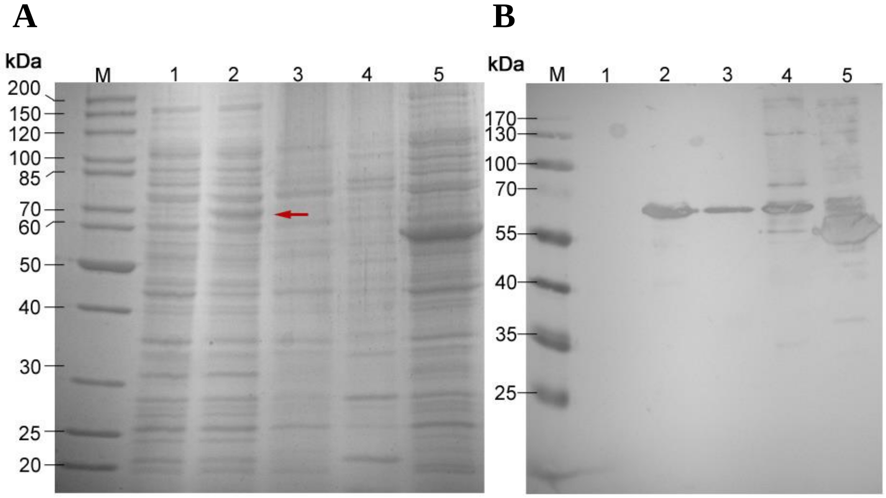

Supplementary Figure S1. Recombinant expression of HFBI-BMO in *E. coli* Shuffle T7 Express. **(A)** SDS-PAGE; **(B)** immunodetection. 1: *E. coli* cells before induction; 2: *E. coli* cells 4 h post-induction; 3: soluble protein fraction 6 h post-induction; 4: insoluble protein fraction 6 h post-induction; 5: reference sample of soluble protein fraction of non-fused BMO (57 kDa). The red arrow marks HFBI-BMO (66 kDa). 10 µg total protein was loaded per lane. M: Molecular weight marker.

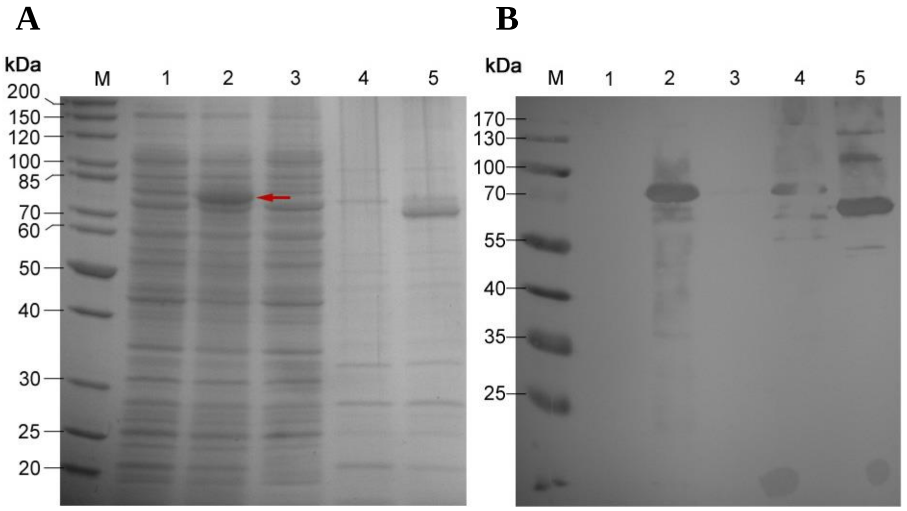

Supplementary Figure S2. Recombinant expression of HFBI-BMR in *E. coli* Shuffle T7 Express. **(A)** SDS-PAGE; **(B)** immunodetection. 1: *E. coli* cells before induction; 2: *E. coli* cells 4 h post-induction; 3: soluble protein fraction 6 h post-induction; 4: insoluble protein fraction 6 h post-induction; 5: reference sample of soluble protein fraction of non-fused BMR (68 kDa). The red arrow marks HFBI-BMR (76 kDa). 10 µg total protein was loaded per lane. M: Molecular weight marker.

1.1.2 Expression of BMO and BMR

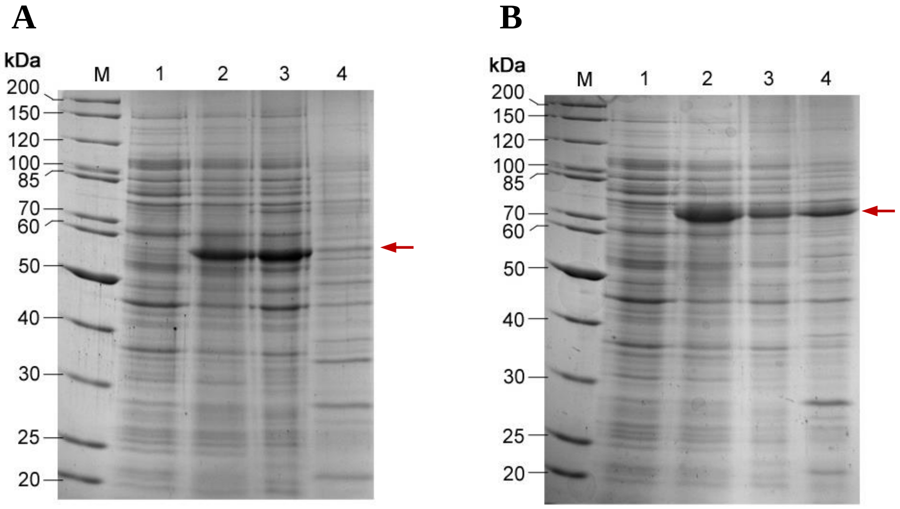

Supplementary Figure S3. SDS-PAGE of **(A)** BMO and **(B)** BMR recombinant expression in *E. coli* Shuffle T7 Express. 1: *E. coli* cells before induction; 2: *E. coli* cells 4 h post-induction; 3: soluble protein fraction 6 h post-induction; 4: insoluble protein fraction 6 h post-induction. The red arrows mark BMO (57 kDa) and BMR (68 kDa). 10 µg total protein was loaded per lane. M: Molecular weight marker.

1.1.3 Myristic acid conversion

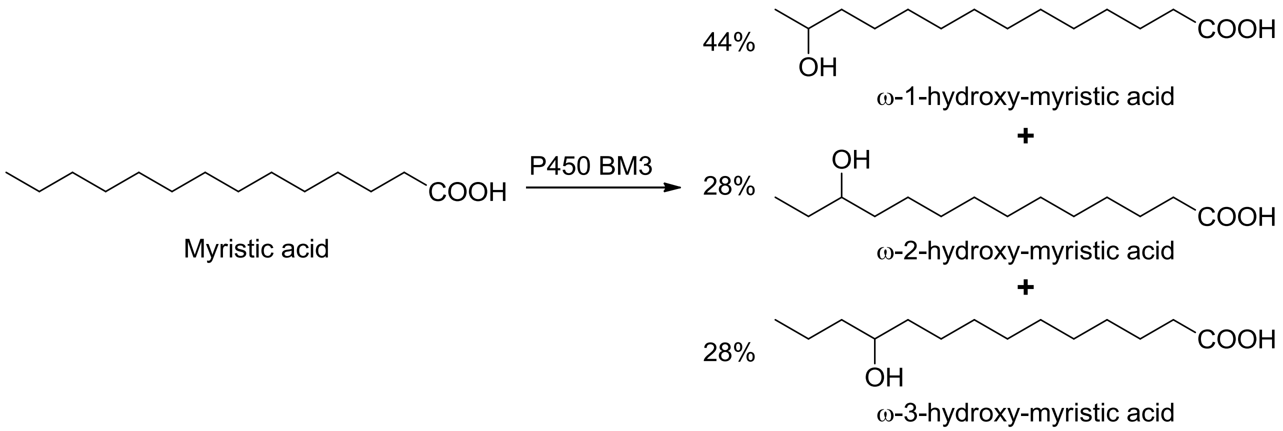

Supplementary Figure S4. Reaction products of myristic acid conversions by the holoenzyme P450 BM3; percentaged product distribution according to (Whitehouse et al., 2012).

1.2 Supplementary Tables

Supplementary Table S1. Synthetic oligonucleotides used for PCR. Start (ATG) and stop (TAA) codons are highlighted in bold characters, restriction sites - in italics and underlined, and the inserted linker sequences - in small characters.

No.	Name	Sequence (5‘ - 3‘)	Restriction site	Application
1	*mhfbI_Eco*RI_ fwd	GTGCGA*GAATTC***ATG**AGCAATG GTAATGGCAATGTTTGTC	*Eco*RI (5‘)	*hfb1-Linker*, N-terminal fusion
2	*mhfbI_Xho*I_ rev	AGATTA*CTCGAG***TTA**TGCACCA ACTGCGGTCTG	*Xho*I (3‘)	*Linker-hfb1*, C-terminal fusion
3	*mhfbI*_Linker_ rev	agagccaccacctccagatccaccgccaccTGC ACCAACTGCGGTCTG	-	*hfb1-Linker* N-terminal fusion
4	*mhfbI*_Linker_ fwd	ggtggcggtggatctggaggtggtggctctATG AGCAATGGTAATGGCAA	-	*Linker-hfb1*, C-terminal fusion
5	*BMO*_Linker_ fwd	ggtggcggtggatctggaggtggtggctctATG ACAATTAAAGAAATGCC	-	*Linker-bmo*, N-terminal fusion
6	*BMO_Xho*I_rev	GCACAG*CTCGAG***TTA**GCGTACT TTTTTAGCAGACTG	*Xho*I (3‘)	*Linker-bmo*, N-terminal fusion
7	*BMR*_Linker_ fwd	ggtggcggtggatctggaggtggtggctctATG AAAAAGGCAGAAAACGC	-	*Linker-bmr*, N-terminal fusion
8	*BMR_Xho*I_rev	AGATTA*CTCGAG***TTA**CCCAGCC CACACGTCTTTTGC	*Xho*I (3‘)	*Linker-bmr*, N-terminal fusion
9	*BMO_Eco*RI_ fwd	GTGCGA*GAATTC***ATG**ACAATTA AAGAAATGCCTCAGCC	*Eco*RI (5‘)	*bmo-Linker*, C-terminal fusion
10	*BMO*_Linker_ rev	agagccaccacctccagatccaccgccaccGC GTACTTTTTTAGCAGAC	-	*bmo-Linker*, C-terminal fusion
11	*BMR_Eco*RI_ fwd	GTGCGA*GAATTC***ATG**AAAAAGG CAGAAAACGCTC	*Eco*RI (5‘)	*bmr-Linker*, C-terminal fusion
12	*BMR*_Linker_ rev	agagccaccacctccagatccaccgccaccCCC AGCCCACACGTCTTTTG	-	*bmr-Linker*, C-terminal fusion

Supplementary Table S2. Temperature profile for PCRs for the amplification of genes containing a linker sequence. The order of the gene names corresponds to their sequential arrangement within a construct.

Step	Number of cycles	Resulting constructs					
		hfb1-Linker; Linker-hfb1		*bmo-Linker; bmr-Linker; Linker-bmo*		*Linker-bmr*	
		Temp. [°C]	Time	Temp. [°C]	Time	Temp. [°C]	Time
**1. Initial Denaturation**	1	98	30’’	98	30’’	98	30’’
**2. Denaturation**	30	98	10’’	98	10’’	98	10’’
**3. Annealing**		59	15’’	58	15’’	61	15’’
**4. Extension**		72	10’’	72	55’’	72	54’’
**5. Final Extension**	1	72	2’	72	5’	72	5’

Supplementary Table S3. Temperature profile for one-step overlap-extension PCR to generate the C-terminal fusion constructs *bmo-hfb1* and *bmr-hfb1*. The order of the gene names corresponds to their sequential arrangement within a construct.

Step	Number of cycles	*bmo-hfb1*		*bmr-hfb1*	
		Temp. [°C]	Time	Temp. [°C]	Time
**1. Initial Denaturation**	1	98	30‘‘	98	30’’
**2. Denaturation**	35	98	10’’	98	10’’
**3. Annealing**		60	15’’	60	15’’
**4. Extension**		72	52’’	72	62’’
**5. Final Extension**	1	72	5’	72	5’

Supplementary Table S4. Temperature profile for two-step overlap-extension PCR to generate the N-terminal fusion constructs *hfb1-bmo* and *hfb1-bmr*. The order of the gene names corresponds to their sequential arrangement within a construct.

Step	Number of cycles	*hfb1-bmo*		*hfb1-bmr*	
		Temp. [°C]	Time	Temp. [°C]	Time
1. Initial Denaturation	1	98	30‘‘	98	30’’
2. Denaturation	7	98	10’’	98	10’’
3. Annealing		60	15’’	60	15’’
4. Extension		72	52’’	72	62’’
5. Final Extension	1	72	5’	72	5’
6. Initial Denaturation	1	98	30’’	98	30’’
7. Denaturation	28	98	10’’	98	10’’
8. Annealing		60	15’’	60	15’’
9. Extension		72	52’’	72	62’’
10. Final Extension	1	72	5’	72	5’

Supplementary Table S5. Product distributions of myristic acid conversions determined after 24 h in reconstituted P450-reductase systems (5-fold molar excess of reductase).

Reconstituted system	Product distribution [%]		
	ω-1	ω-2	ω-3
BMO-HFBI / BMR-HFBI	47	25	28
BMO / BMR	42	26	32

2 Supplementary references

Whitehouse, C.J.C., Bell, S.G., and Wong, L.L. (2012). P450(BM3) (CYP102A1): connecting the dots. *Chemical Society reviews* 41(3)**,** 1218-1260. doi: 10.1039/c1cs15192d.
